# Relationship of Ultrasonically Assessed Diaphragmatic Weakness with the Need for Postoperative Mechanical Ventilation in Older Adult Patients after Major Abdominal Surgeries: An Observational Study

**DOI:** 10.4274/TJAR.2026.262608

**Published:** 2026-08-28

**Authors:** Jyoti Suthar, Deepak Singla, Bhavna Gupta, Sanjay Agrawal, Mridul Dhar, Ranjeeta Kumari

**Affiliations:** 1 All India Institute of Medical Sciences, Department of Anaesthesia and Critical Care, Rishikesh, India; 2 All India Institute of Medical Sciences, Department of Community and Family Medicine, Rishikesh, India

**Keywords:** Assessment, diaphragm, general anaesthesia, older adult, postoperative complications, ultrasonography

## Abstract

**Objective:**

This study aimed to analyze the association between preoperative diaphragmatic weakness in older adult patients and the need for postoperative mechanical ventilation after major surgery. We hypothesized that preoperative diaphragmatic weakness would significantly increase the need for postoperative mechanical ventilation.

**Methods:**

It was a single-center, prospective, observational study that included 90 older adult patients aged more than 50 years who underwent abdominal surgery under general anaesthesia. A portable ultrasound was used to assess the percentage increase in the diaphragm thickening fraction (TFdi) during a maximal inspiratory effort, preoperatively and before extubation. The perioperative change in diaphragmatic function, the development of outcomes, i.e., extubation/mechanical ventilation, and the optimal cut-off of TFdi to predict outcomes were analyzed.

**Results:**

The patients with a preoperative TFdi <35% had a higher requirement for mechanical ventilation (24.5% or 1:3) as compared to those with TFdi ≥35% (7.3% or 1:13). In multivariate linear regression analysis, TFdi (preoperative) was the only independent predictor of TFdi (before extubation), explaining a substantial proportion of the variance (adjusted R^2^=0.71). The optimal cut-off value of TFdi (preoperative) ≤28.9 predicted intensive care unit stay with 73% sensitivity and 73% specificity, and a high negative predictive value (93.2%).

**Conclusion:**

Our study found that a significant percentage of older adult patients had a preexisting diaphragmatic weakness that was associated with extubation failure and the need for postoperative mechanical ventilation.

**Main Points**

• Approximately half of the geriatric patients posted for major surgeries had diaphragmatic weakness before surgery.

• There was a statistically significant high association between preoperative and postoperative diaphragmatic weakness.

• The patients with poor diaphragm function in the preoperative period had a larger proportion of patients with higher requirement for mechanical ventilation.

• The preoperative ultrasound based diaphragm assessment was able to predict the need for postoperative mechanical ventilation with a high negative predictive value.

## Introduction

Primary sarcopenia is an age-related reduction in muscle mass.^1^ If respiratory muscles, along with skeletal muscles, are involved, it is referred to as respiratory sarcopenia. Since it is a common finding in the older-adult population, it has been named “presbypnea,” in which the prefix “presby-” means older adult and the suffix “-pnea” refers to respiration.^2^ A reduction in transdiaphragmatic pressure of 20-41% and an overall reduction in respiratory muscle strength of 30% with age have been observed.^3^ Factors that contribute to the pathology of diaphragmatic muscle weakness include undernutrition, cachexia, aspiration pneumonia, and chronic obstructive pulmonary disease (COPD).^4^

Studies have shown that, in critically ill patients, the diaphragm can be significantly weakened postoperatively because it is fatigued during invasive mechanical ventilation.^5^ A systematic review by Powers et al.^6^ in 2010 concluded that as little as 18 hours of mechanical ventilation can cause significant diaphragmatic atrophy. A fatigued diaphragm is associated with difficulty with extubation, weaning failure, increased risk of readmission, and increased 1-year post-surgery mortality.^7, 8^ Since diaphragmatic weakness can be a significant factor determining postoperative outcomes in elderly patients, it should be assessed using reliable methods to improve postoperative outcomes and to provide rehabilitation when necessary.

Ultrasound assessment of the diaphragm is a feasible modality due to the widespread availability of portable ultrasound. Various ultrasound-based approaches are available for diaphragmatic assessment. In the ABC Diaphragmatic Evaluation (ABCDE) approach, the percentage change in diaphragm thickness reflects the degree of effort exerted by the muscle. In normal individuals, the change ranges from 28-96%; in individuals with diaphragmatic paralysis, it ranges from 5-35%.^9^ The ABCDE approach is a relatively new, less observer-dependent, and easy-to-perform method that allows more straight forward quantification of diaphragmatic weakness.

Despite a thorough literature search, we were unable to identify any study in which diaphragm function was assessed before surgery in older adult patients. This study aimed to analyze the association between diaphragmatic weakness in older adult patients scheduled for abdominal surgeries under general anaesthesia and the need for postoperative mechanical ventilation. We hypothesized that preoperative diaphragmatic weakness would significantly increase the need for postoperative mechanical ventilation.

## Methods

### Study Participants

This study is a prospective observational study. It was conducted after receiving approval from the Ethics Committee of the All India Institute of Medical Sciences in Rishikesh, India (approval no: AIIMS/EC/23/501, date: 08.12.2023). The study is registered in the Indian Clinical Trials Registry (CTRI/2025/05/087124). For this study, we followed the Helsinki Declaration of 1964, updated in 2013. The study was conducted in accordance with the Strengthening the Reporting of Observational Studies in Epidemiology guidelines. The inclusion criteria for our study were patients aged more than 50 years, of any sex, classified as American Society of Anesthesia (ASA) class I to III, and undergoing abdominal surgery under general anaesthesia. The exclusion criteria were refusal to consent for the procedure, patients with injury to chest or diaphragm or surgery of chest/diaphragm, severely compromised lung function i.e., severe COPD (GOLD class 3, 4), moderate to severe pulmonary arterial hypertension, acute respiratory distress syndrome with PaO_2_/FiO_2_ <150, any known cardiac disease (ischemic heart disease, valvular heart disease, congestive heart failure), altered Glasgow Coma Scale, already on mechanical ventilation, home oxygenation/non-invasive ventilation and those in shock mean arterial pressure (MAP)<60 mm of Hg or requiring inotropes to maintain MAP>65 mm of Hg.

### Diaphragm Assessment

The study protocol was explained to the patients. Written consent was obtained from those willing to participate in our study. After confirming adequacy of nil per oral status, patients were taken into the preoperative room; routine monitors [i.e., pulse oximeter, electrocardiogram (ECG), and non-invasive blood pressure (NIBP)] were attached, and the Mini Nutritional Assessment (MNA) score was calculated. Diaphragm function was assessed using the portable ultrasound machine (SonoSite Edge II Ultrasound System, Fujifilm inc.). Ultrasound was used to assess the percentage increase in diaphragm thickening fraction (TFdi) during a maximal inspiratory effort using the ABCDE approach.^9^ In this approach, the patient is positioned supine. A linear high-frequency (5-13 MHz) ultrasound probe was used with a portable ultrasound machine (SonoSite Edge II Ultrasound System, Fujifilm inc.). The linear probe was placed on the anterior axillary line at the level of the nipple, first on the patient’s right side. The probe slid downward until the diaphragm was visible during both inspiration and expiration. The change in diaphragm thickness was assessed during maximal inspiratory effort in a spontaneously breathing patient. As the diaphragm contracted during inspiration, diaphragm thickness increased. That was assessed in M-mode ultrasound and expressed as the percentage increase in thickness (TFdi). If the change in thickness was >35%, it was regarded as normal diaphragmatic function; if it was <35%, it was labelled as diaphragmatic weakness.^9^ All assessments were performed by a single anaesthesiologist who was not involved in patient management. All the ultrasound scans were performed by a single operator in our study. The operator was well trained in ultrasound assessment of the diaphragm and had already performed more than 100 ultrasound examinations in both the intensive care unit and the operation theatre.

Patients were then taken to the operating theatre. All the routine monitors, i.e., pulse oximeter, ECG, and NIBP, were attached. In addition to routine monitors, we connected the train-of-four (TOF) ratio and the bispectral index monitor to the patient. An epidural catheter was inserted at the discretion of the anaesthesiologist. Patients were preoxygenated, followed by administration of injection (inj.) fentanyl 1-2 μg kg^-1^, propofol (1-2.5 mg kg^-1^), or etomidate (0.2-0.3 mg kg^-1^). Vecuronium 0.1 mg kg^-1^ i.v. was used. Patients were intubated and mechanically ventilated. The anaesthesia was maintained using sevoflurane with 40% oxygen. Inj. Vecuronium 0.02 mg kg^-1^ i.v. was given if a single twitch appeared on the TOF monitor. The total maintenance dose of inj. vecuronium was calculated.

After surgery, anaesthetic agents and muscle relaxants were discontinued. Inj. Neostigmine 0.35-0.5 mg kg^-1^ iv was administered. As the patient regained spontaneous respiratory efforts, they were switched to pressure support mode. Pressure support was gradually reduced, and when support reached 6-8 cm of H_2_O with positive end-expiratory pressure of 5 cm of H_2_O, diaphragm assessment (TFdi) was repeated. A lung ultrasound score was calculated for all patients to determine their lung condition and to assess the need for mechanical ventilation based on that score. We followed the BLUE protocol for postoperative lung ultrasound.

Patients were extubated when they met clinical criteria for extubation (i.e., regular respiration, stable vital signs, ability to follow commands, adequate hand grip, and ability to lift the head). Any patient not meeting the criteria was considered for elective mechanical ventilation at the discretion of the treating anaesthesiologist and the surgeon. All patients were transferred to the post-anaesthesia care unit, the intensive care unit, or the high-dependency unit, depending on ventilator availability.

### Objectives and Variable Definitions

The primary objective was to determine the percentage of older adult patients with preoperative diaphragmatic weakness, as assessed by ultrasound, who required postoperative mechanical ventilation after abdominal surgery under general anaesthesia. The secondary objectives were to determine the perioperative change in diaphragmatic function (i.e., TFdi: preoperative versus before extubation); to assess the relationship between preoperative and intraoperative variables and diaphragmatic function; to evaluate the association of diaphragmatic function with outcomes (i.e., extubation and postoperative mechanical ventilation); and to determine the optimal cut-off of TFdi at the preoperative and before-extubation time points to predict extubation/postoperative mechanical ventilation.

### Sample Size Calculation

Since we could not find any similar studies in the literature, we conducted a pilot study of 20 older adult patients to inform sample size calculation and found the prevalence of postoperative mechanical ventilation in patients with pre-operative diaphragmatic weakness, as assessed by ultrasound, to be 85% (13 patients had TFdi<35% and 11 required mechanical ventilation). Based on that, the sample size was calculated using the following formula:

n = Z2_1-a/2_ pq/L2

where: n = required sample size, Z (0.05)=1.96 (value from normal standard deviation (SD) reflecting the 95% confidence interval), p (prevalence rate)= 85%, q=1-p=15%, L=10% [least permissible error(absolute precision)]

Hence sample size =

A total of 49 patients with preoperative diaphragm weakness were required for our study. All older adult patients were recruited until we included a total of 49 patients with diaphragmatic weakness. A total of 90 patients were included in our study.

### Statistical Analysis

Data were coded and recorded in a Microsoft Excel spreadsheet. The Statistical Package for the Social Sciences, version 23 (IBM Corp.), was used for data analysis. The Mann-Whitney U test was applied to skewed or non-normally distributed data to compare continuous variables between two independent groups. To assess associations between categorical variables, the chi-squared test was used. In cases where expected cell counts were less than 5 in more than 20% of the cells, Fisher’s Exact test was applied instead. The strength of association between the two variables was measured using Cramer’s V or the point-biserial correlation. Multivariate linear regression with an omnibus ANOVA was used to analyze the association between TFdi (preoperative) and preoperative variables, and between TFdi (before extubation) and intraoperative variables. receiver operating characteristic (ROC) curves were used to assess the diagnostic performance of a variable and to determine its optimal cut-off value. A *P* value <0.05 was considered statistically significant.

## Results

### Study Participants

**Figure 1:**
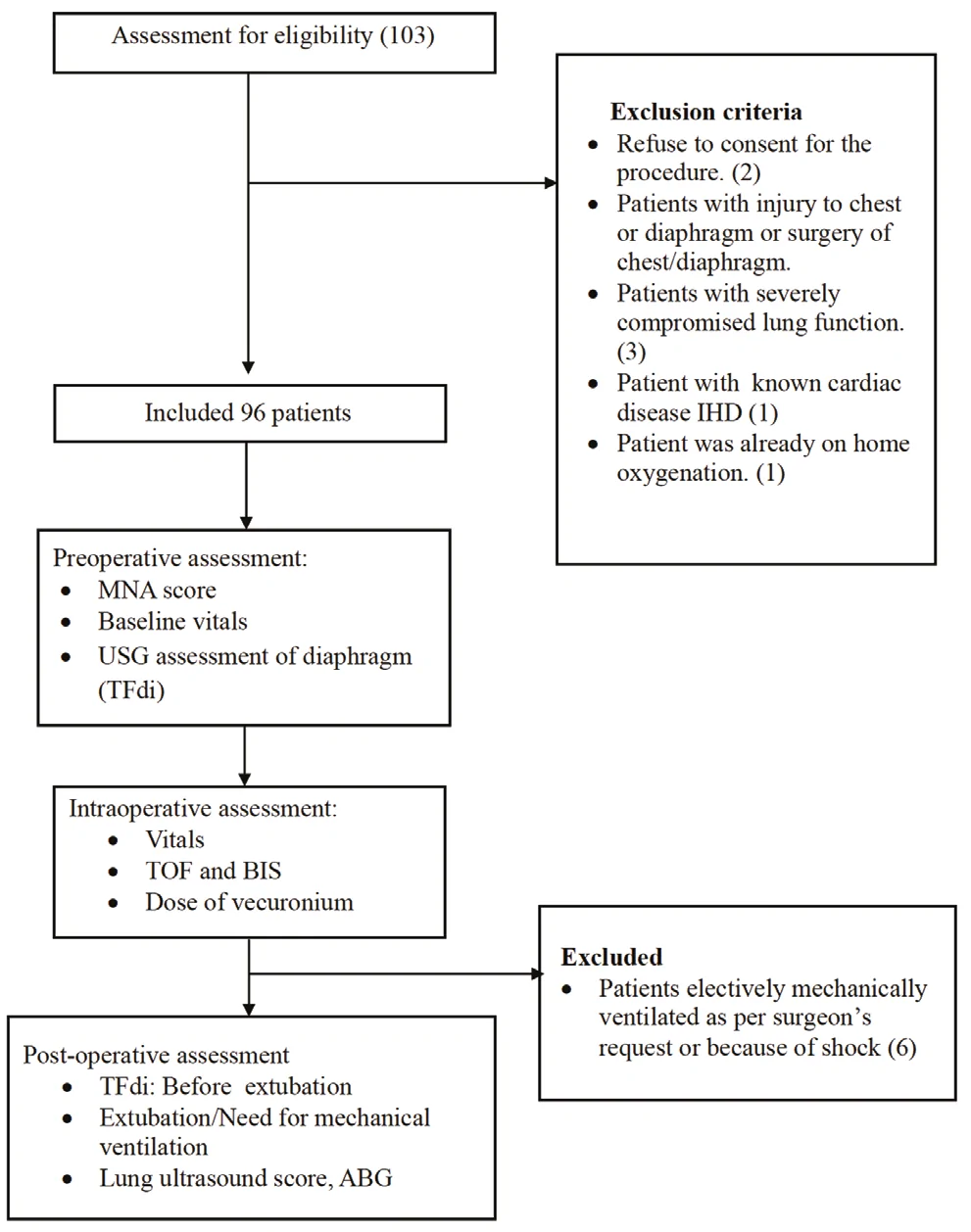
Methodology flowchart. MNA, Mini Nutritional Assessment; TFdi, thickening fraction; BIS, bispectral index; TOF, train-of-four; USG, ultrasonography; ABG, arterial blood gas; IHD, ichemic heart disease.

We considered 103 patients for inclusion in our study. Of these, two patients refused to consent to the procedure, three patients had severely compromised lung function, one patient had known cardiac disease, and one patient was on home oxygenation (Figure 1). A total of 96 patients were included in our study. However, six patients required postoperative mechanical ventilation—either at the request of the surgical team or because of severe shock—so reversal of muscle relaxation was not performed in these patients immediately after surgery, and they were excluded from the study. The remaining 90 patients received a trial of extubation and were thus included in our study (Figure 1).

The demographic profile of the patients is described in Table 1. Most patients underwent major abdominal, gynecological, or urological surgeries, as shown in Supplementary Table 1. The mean duration of surgery was 260.22 (109.02) minutes, and the mean duration of anaesthesia was 308.89 (124.34) minutes (Table 1).

### Diaphragm Assessment

Forty-nine (54.4%) participants at the pre-operative time point and 66 (73.3%) before extubation had TFdi <35%. The mean (SD) TFdi (%) in the study population decreased from 36.84 (16.13) preoperatively to 29.84 (14.64) before extubation (Table 2). This change was statistically significant (Friedman test: χ^2^=79.4, *P≤*0.001). The association between TFdi (pre-operative) and TFdi (before extubation) is shown in Table 2. Participants in the TFdi group (pre-operative: <35%) had the larger proportion of TFdi before extubation (<35%). The strength of association between TFdi (pre-operative) and TFdi (before extubation), as measured by Cramer’s V, was 0.56 (high association).

### Relationship of Peri-Operative/Intraoperative Variables and Diaphragm Weakness

The multivariate linear regression analysis of TFdi (preoperative) and preoperative variables is shown in Supplementary Tables 2A-C. On analyzing the overall model performance, the R was 0.36, suggesting weak correlation with only 13% variability (R^2^). The overall predictive ability of the model was poor. In the omnibus ANOVA, the MNA score had a *P* value of 0.032. The regression coefficient for the MNA score was estimated as 1.76 (95% CI: 0.15 to 3.36). For every 1 point increase in the MNA score, the TFdi (pre-operative) increases by ~1.76 units, after adjusting for all other variables.

For TFdi (before extubation), the multivariate linear regression analysis yielded R=0.87, indicating an excellent overall correlation and explaining 73% of the variance (Supplementary Tables 3A-C. In the omnibus ANOVA, TFdi (pre-operative) was significantly associated with TFdi (before extubation) (*P* < 0.001). For every 1% increase in TFdi (pre-operative), the TFdi (before extubation) increases by 0.77%, independent of perioperative and physiological variables.

### Association of Diaphragm Weakness and Outcomes

The association between TFdi and outcomes such as extubation and postoperative mechanical ventilation was examined in Table 3. Significant differences in the distribution of patient outcomes were observed between the groups. 75.5% of the participants in the group TFdi (pre-operative) <35% had extubation while 92.7% of the participants in the group TFdi (pre-operative): ≥35% had extubation (Table 3). The Strength of association between the two variables (Cramer’s V)=0.23 (low association). 77.3% of the participants in the group TFdi (before extubation) <35% had extubation while 100.0% of the participants in the group TFdi (before extubation) ≥35% had extubation (Table 3). The strength of association between the two variables (Cramer’s V) was 0.27, indicating a low association.

### ROC Curve for TFdi Pre-Operative and Before Extubation

**Figure 2:**
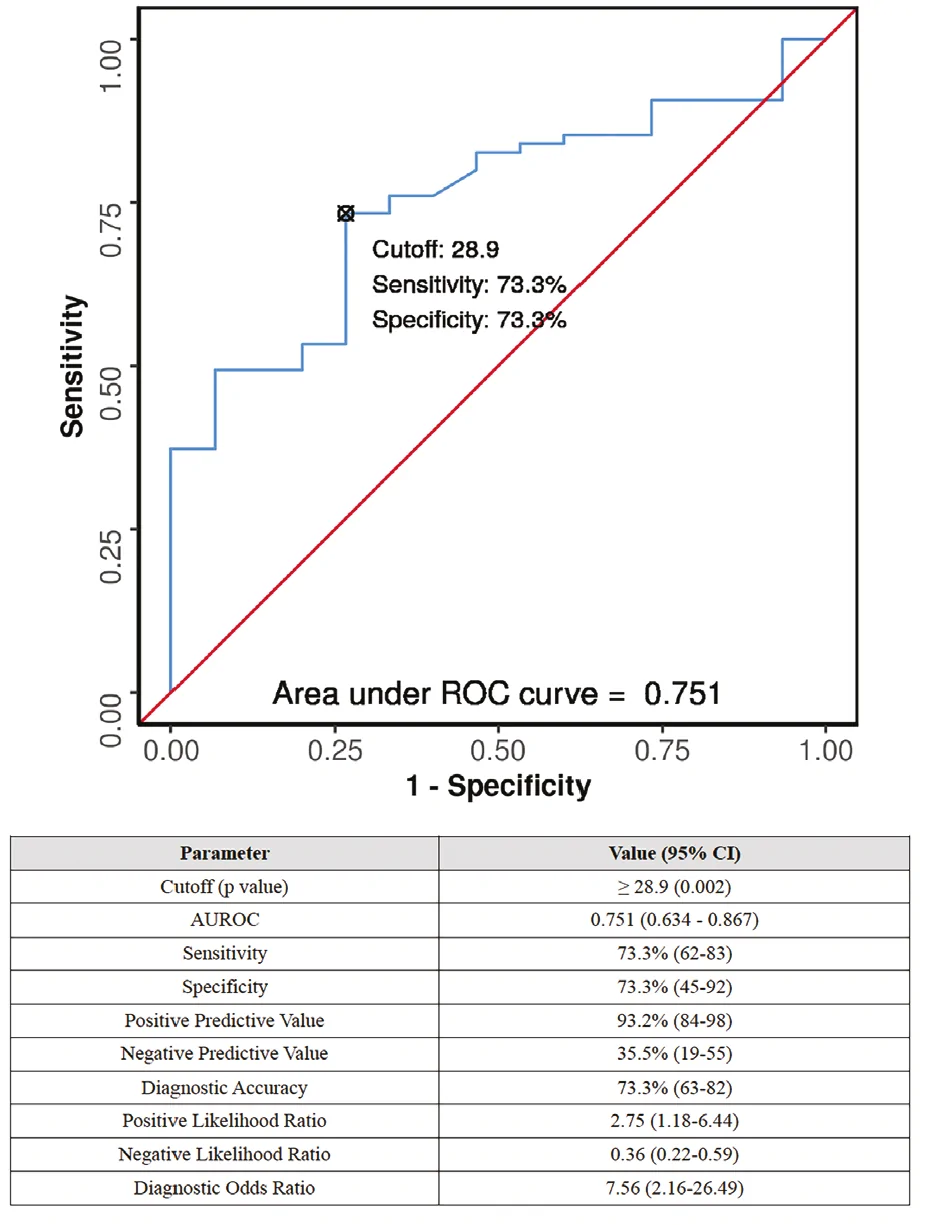
ROC curve analysis showing diagnostic performance of TFdi (%) (pre-operative) in predicting extubation: yes vs. extubation: no (n = 90). ROC, receiver operating characteristic; TFdi, diaphragm thickening fraction.

**Figure 3:**
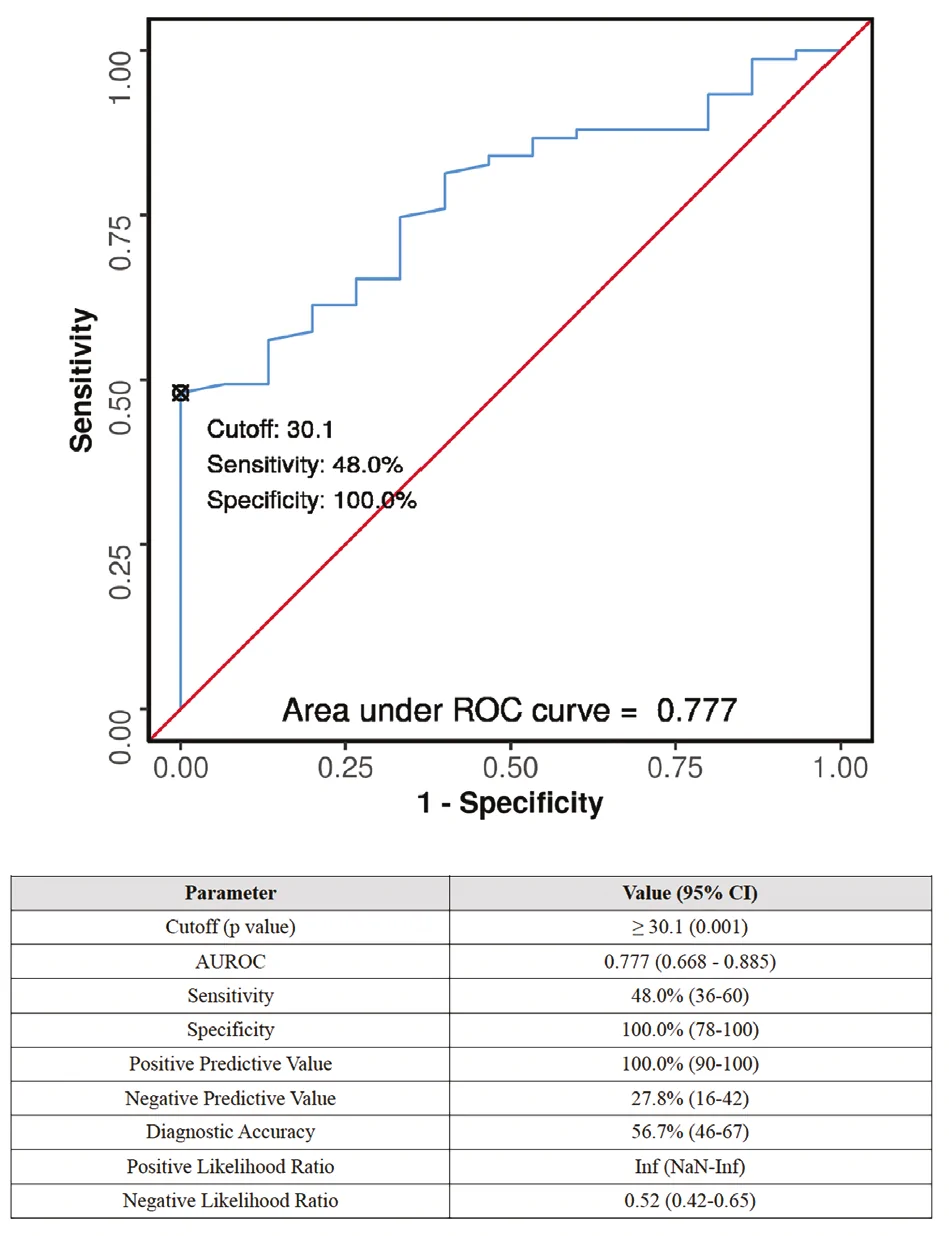
ROC curve analysis showing diagnostic performance of TFdi (%) (before extubation) in predicting extubation: yes vs. extubation: no (n = 90). ROC, receiver operating characteristic; TFdi, diaphragm thickening fraction.

The area under the ROC curve (AUROC) for pre-operative TFdi (%) predicting extubation was 0.751 (95% CI: 0.634-0.867), indicating fair diagnostic performance (Figure 2). This result was statistically significant (*P*=0.002). At a cut-off of TFdi (pre-operative) ≥28.9, it predicts extubation: Yes, with a sensitivity of 73%, and a specificity of 73%. For TFdi (before extubation) predicting extubation (yes vs no), the AUROC was 0.777 (95% CI: 0.668- 0.885), indicating fair diagnostic performance (Figure 3). It was statistically significant (*P* = 0.001). At a cut-off of TFdi (before extubation) ≥30.1, it predicts extubation: Yes, with a sensitivity of 48%, and a specificity of 100%. However, 100% specificity may be due to a small number of events.

### Predictive Ability of Pre-Operative Variables for Postoperative Outcome

We performed a binomial logistic regression analysis to assess whether combinations of preoperative variables can predict postoperative extubation or the need for mechanical ventilation (Supplementary Table 4A, 4B and Tables 4A-C, Supplementary Figure 1). The model demonstrated good overall fit. ASA class III status, lower MNA scores, and higher BMI were significantly associated with increased odds of postoperative mechanical ventilation. Preoperative TFdi showed a borderline association, which did not reach statistical significance. Overall, the model demonstrated accuracy of 90%, specificity of 96%, and sensitivity of 60%. An AUC of 0.93 indicated outstanding discrimination between patients likely to be extubated and those requiring postoperative mechanical ventilation.

## Discussion

We conducted an observational study of 90 older adult patients. We found that the numbers (percentages) of patients with TFdi values below 35% were 49 (54.4%) in the preoperative period and 66 (73.3%) just before extubation. In multivariate linear regression analysis, the preoperative MNA score was the only variable independently associated with TFdi (preoperative), whereas TFdi (preoperative) was the only independent predictor of TFdi (before extubation), explaining a substantial proportion of the variance (adjusted Rz^2^=0.71). The patients with a preoperative TFdi <35% had a higher requirement for mechanical ventilation (24.5% or 1:3) as compared to those with TFdi ≥35% (7.3% or 1:13). The optimal cut-off value of TFdi (preoperative) ≤28.9 predicted need for postoperative mechanical ventilation with 73% sensitivity and 73% specificity.

In our study, we found that the number and percentage of patients with TFdi values below 35% were 49 (54.4%) in the preoperative period and 66 (73.3%) just before extubation. We also found a significant reduction in mean TFdi values from the preoperative level (36.84) to just before extubation (29.84). Moury et al.^10^ conducted a similar study in which they tested for TFdi in patients undergoing elective cardiac surgery. The mean (SD) preoperative value of TFdi was 36 (18) and decreased to 17 (14) during a spontaneous breathing trial (SBT) and 12 (11) on day +1 (*P *< 0.0001). In a study by Dres et al.,^11^ the median TFdi of all subjects was 28%. Patients who passed SBT had higher TFdi values than those who failed SBT (33% vs. 19%). Lee et al.^12^ conducted a study in pediatric patients, in which diaphragm thickness fraction (DTF) decreased markedly during the first 24 hours of mechanical ventilation.

In multivariate linear regression analysis, the pre-operative MNA score was the only variable significantly associated with TFdi (preoperative), whereas TFdi (preoperative) was the only independent predictor of TFdi (before extubation) and explained a substantial proportion of the variance (adjusted R^2^=0.71). Uyar et al.^13^ studied the mechanically ventilated intensive care unit (ICU) patients. They concluded that high protein intake (2.0 g kg^-1^ day^-1^) significantly increased diaphragm and rectus femoris muscle thickness compared to standard protein intake, suggesting that high protein support may help prevent muscle atrophy in critical care.

In our study, TFdi was significantly associated with patient extubation or need for mechanical ventilation, both preoperatively (χ^2^=4.740, *P*=0.029) and before extubation (χ^2^=6.545, *P*=0.009). However, the strength of the association was low in both cases. In the study by Lee et al.^12^ the DTF% of less than 17% was significantly correlated with extubation failure (*P *< 0.001). Cammarota et al.^14^ found that patients who fail extubation had significantly increased diaphragmatic activation during SBT. Vivier et al.^15^ found that the diaphragm excursion measured by bedside ultrasound along with airway pressure assessment was more accurate than electromyography in detecting patient ventilator asynchrony. However, studies by and Vetrugno et al.^16^ reported that diaphragmatic excursion and thickening fraction were not reliable for predicting extubation and weaning failure, including in coronavirus disease-2019 patients. These results may be due to poor lung compliance and a low P/F ratio among the study patients.

In our study, the optimal cut-off value of TFdi (preoperative) ≥28.9 predicted extubation with 73% sensitivity and 73% specificity, and a high negative predictive value (93.2%), which indicates that patients with TFdi above this threshold are unlikely to require mechanical ventilation. In the study by Moury et al.,^10^ the decrease in TFdi was correlated with increased length of stay in the ICU. The diaphragm thickening fraction (TFdi), measured by ultrasound, was below 20% in 70% of patients, indicating reduced diaphragmatic activity. Their ultrasound finding showed a moderate correlation with diaphragm strength (r=0.4, *P*=0.02), but it did not correlate with overall muscle strength. In a study by Dres et al.,^11^ a TFdi threshold of 25.8% was identified as predictive of weaning failure (*P* < 0.001). Another study used a reduction of 25% or more in diaphragm excursion during the sniff test as a criterion for hemidiaphragmatic paralysis.^17^

### Study Limitations

Our study had certain limitations. We included patients aged 50 years and older. However, the patient profile might have differed if only patients from an older age group (i.e., >65 years) had been included in our study. Secondly, although all ultrasound scans were performed in our study, the multivariate linear regression model for perioperative TFdi showed only a weak predictive ability, suggesting that there may be other factors, such as neuromuscular function and cardiopulmonary reserve, which were not analyzed in our study. Thirdly, several other perioperative factors independent of diaphragmatic function—such as epidural analgesia, residual neuromuscular blockade, opioid exposure, pulmonary comorbidities, frailty, nutritional impairment, and lung ultrasound findings—might have influenced the need for mechanical ventilation. Although we have briefly touched upon some of these factors, a detailed analysis of all of them was beyond the scope of this study. Further studies are required to evaluate the effect of adding an epidural to general anaesthesia. The sample size may have been insufficient for specific secondary objectives; therefore, further studies with larger sample sizes may be considered.

## Conclusion

Our study found that a significant percentage of older adult patients scheduled for major elective abdominal procedures had a pre-existing diaphragmatic weakness that worsened further in the postoperative period. Lower preoperative TFdi was significantly associated with extubation failure and the need for postoperative mechanical ventilation. Better nutritional status was linked to higher TFdi values, indicating a possible relationship between nutrition and diaphragm strength. TFdi is a valuable and non-invasive indicator of diaphragmatic function and can help predict the need for postoperative mechanical ventilation in elderly patients.

## Ethics

**Ethics Committee Approval:** It was conducted after receiving approval from the Ethics Committee of the All India Institute of Medical Sciences in Rishikesh, India (approval no: AIIMS/EC/23/501, date: 08.12.2023).

**Informed Consent:** This study is a prospective observational study and therefore exempts patient consent.

## Supplementary Materials

Supplementary Materials https://d2v96fxpocvxx.cloudfront.net/ae7a798f-afa1-42f4-9efd-6a2ed974293f/content-images/053a3bdd-435d-4864-b14b-89db6bb19517.pdf

## Figures and Tables

**=:**
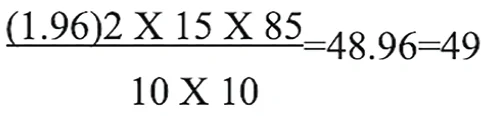


**Table 1. Demographic Profile of the Patients Included in the Study:** 

**Basic details**	**Mean (SD)**	**Median (IQR)**	**Min-max OR n (%)**
**Age** **(years)**	63.7 (6.6)	64.0 (58.3-68.7)	52.0-80.0
**Age**			
50-59 years	27 (30.0%)	-	-
60-69 years	44 (48.9%)	-	-
70-79 years	18 (20.0%)	-	-
80-89 years	1 (1.1%)	-	-
**Gender**		-	-
Male	36 (40.0%)	-	-
Female	54 (60.0%)	-	-
**Weight** **(kg)**	59.2 (10.5)	58.5 (52.0-68.0)	38.0-88.0
**Height** **(cm)**	160.7 (8.0)	159.0 (155.3-166.0)	144.0-182.0
**BMI** **(kg/m^2^)**	23.1 (3.8)	23.2 (20.7-25.6)	15.0-34.3
**ASA**			
I	25 (27.8%)	-	-
II	53 (58.9%)	-	-
III	12 (13.3%)	-	-
**Anaesthesia**		-	-
GA	28 (31.1%)	-	-
GA+EA	62 (68.9%)	-	-

SD, standard deviation; IQR, interquartile range; BMI, body mass index; ASA, American Society of Anesthesiologists; GA, general anaesthesia; EA, epidural anaesthesia; OR, odds ratio; Min-max, minimum-maximum

**Table 2. Assessment of “TFdi (pre-operative)” & “TFdi (before extubation)” and the Association of “TFdi (pre-operative)” & “TFdi (before extubation)”:** 

**Timepoint**	**TFDI (%)**			**Mann-Whitney U**		**Rank biserial correlation**
	**Mean (SD)**	**Median (IQR)**	**Range**	**Static**	***P* value**	
**Pre-operative**	36.8 (16.1)	33.4 (21.6)	9.09-80.00	2943.50	0.002	-0.27
**Before** **extubation**	29.8 (14.6)	27.53 (18.9)	6.37-76.00			
**TFdi**	**<35%**	**≥35%**	**TFdi** **(before** **extubation)**	**Chi-squared** **test** **(χ^2^)**	***P*** **value**	**Cramer’s** **V**
<35%	47 (95.9%)	19 (46.3%)	66 (73.3%)	28.056	<0.001	0.56 (high association)
≥35%	2 (4.1%)	22 (53.7%)	24 (26.7%)			
**TFdi** **(pre-operative)**	49 (100%)	41 (100%)	90 (100%)			

SD, standard deviation; IQR, interquartile range; TFdi, diaphragm thickening fraction

**Table 3. Association Between “TFdi (<35%)/TFdi (≥35%)” and “Extubation/Post-Operative Mechanical Ventilation”:** 

**Patient outcome**	**TFdi (pre-operative)**			**Chi-squared test**	
	**<35%**	**≥35%**	**Total**	**χ^2^**	***P* value**
Mechanical ventilation	12 (24.5%)	3 (7.3%)	15 (16.7%)	4.740	0.029
Extubation	37 (75.5%)	38 (92.7%)	75 (83.3%)		
Total	49 (100%)	41 (100%)	90 (100%)		
**Patient** **outcome**	**TFdi** **(before** **extubation)**			**Fisher’s** **exact** **test**	
	**<35%**	**≥35%**	**Total**	**χ^2^**	***P*** **value**
Mechanical ventilation	15 (22.7%)	0 (0.0%)	15 (16.7%)	6.545	0.009
Extubation	51 (77.3%)	24 (100%)	75 (83.3%)		
Total	66 (100%)	24 (100%)	90 (100%)		

TFdi, diaphragm thickening fraction
